# Dot1 binding induces chromatin rearrangements by histone methylation-dependent and -independent mechanisms

**DOI:** 10.1186/1756-8935-4-2

**Published:** 2011-02-03

**Authors:** Iris JE Stulemeijer, Brietta L Pike, Alex W Faber, Kitty F Verzijlbergen, Tibor van Welsem, Floor Frederiks, Tineke L Lenstra, Frank CP Holstege, Susan M Gasser, Fred van Leeuwen

**Affiliations:** 1 Division of Gene Regulation, Netherlands Cancer Institute, Netherlands Proteomics Centre, Plesmanlaan 121, 1066CX Amsterdam, The Netherlands; 2 Friedrich Miescher Institute for Biomedical Research, Maulbeerstrasse 66, 4058 Basel, Switzerland; 3 Department Molecular Cancer Research, University Medical Center Utrecht, Universiteitsweg 100, 3584 CG Utrecht, The Netherlands

## Abstract

**Background:**

Methylation of histone H3 lysine 79 (H3K79) by Dot1 is highly conserved among species and has been associated with both gene repression and activation. To eliminate indirect effects and examine the direct consequences of Dot1 binding and H3K79 methylation, we investigated the effects of targeting Dot1 to different positions in the yeast genome.

**Results:**

Targeting Dot1 did not activate transcription at a euchromatic locus. However, chromatin-bound Dot1 derepressed heterochromatin-mediated gene silencing over a considerable distance. Unexpectedly, Dot1-mediated derepression was established by both a H3K79 methylation-dependent and a methylation-independent mechanism; the latter required the histone acetyltransferase Gcn5. By monitoring the localization of a fluorescently tagged telomere in living cells, we found that the targeting of Dot1, but not its methylation activity, led to the release of a telomere from the repressive environment at the nuclear periphery. This probably contributes to the activity-independent derepression effect of Dot1.

**Conclusions:**

Targeting of Dot1 promoted gene expression by antagonizing gene repression through both histone methylation and chromatin relocalization. Our findings show that binding of Dot1 to chromatin can positively affect local gene expression by chromatin rearrangements over a considerable distance.

## Background

Post-translational modifications of histone proteins are intimately involved in regulation of gene expression [1]. Histone modifications can influence chromatin structure either directly or via proteins that specifically recognize the modified histones [1]. Methylation of histone H3 lysine 79 (H3K79) by Dot1 (also known as KMT4, DOT1L, mDot1 and grappa) is a histone modification that is highly conserved between species [2]. Several studies have linked Dot1 to gene activation. For example, methylated H3K79 is predominantly located in euchromatic regions of the genome [2-8], and Dot1 has been implicated in reactivation of tumor-suppressor genes upon DNA demethylation [9]. Furthermore, in human leukemias bearing chromosomal translocations at the mixed lineage leukemia (*MLL*) or clathrin assembly lymphoid myeloid (*CALM*) genes, mistargeting of DOT1L leads to transcript upregulation [10-13]. These leukemia-associated fusion proteins recruit DOT1L to target genes, with a concomitant increase in H3K79 methylation around the targeted site, upregulation of gene expression, and subsequent development of leukemia [10]. However, other studies have provided support for a repressive function of DOT1L and H3K79 methylation in mammals [14-18], and loss of Dot1 function has been shown to lead to heterochromatin defects [16,19].

In yeast, ~90% of H3K79 is methylated by Dot1 [2]. Methylated H3K79 is mainly found in euchromatin, and absent from heterochromatic regions such as telomeres and the silent mating-type loci [2,3,6]. Loss of Dot1 activity leads to relocalization of Sir2, 3 and 4, the proteins responsible for heterochromatin-mediated gene silencing [2,7,20-28]. In addition, deletion of Dot1 affects histone hypoacetylation and the ordered nucleosome positioning pattern found in repressive chromatin at yeast telomeres [7,29]. These observations suggest that H3K79 methylation prevents non-specific interactions of the Sir proteins with euchromatic nucleosomes, thus promoting Sir protein accumulation in heterochromatic regions [20,22]. The Sir proteins in turn prevent methylation of H3K79 by Dot1 by multiple mechanisms [26,27,30,31].

The idea that H3K79 may act as an anti-binding signal is supported by the observation that binding of Sir3 to chromatin is negatively affected by methylation of H3K79 [2,7,25,27,32-36], and that the presence of Dot1 delays the re-establishment of Sir3-mediated silencing of a previously derepressed (and presumably H3K79 methylated) locus [34,36]. Although Dot1 affects Sir protein targeting and silencing of reporter genes, loss of Dot1 in otherwise unperturbed cells has minor effects on overall gene expression ([37,38] and our unpublished gene expression profiling results). However, disruption of Dot1 in combination with the disruption of additional silencing pathways results in more pronounced silencing defects in reporter genes [24,25,39] and in the native silent mating-type locus *HML***α **[25,34,40], indicating that the role of Dot1 in gene silencing is masked by redundant pathways [24].

The direct and indirect effects of Dot1 on chromatin organization and gene regulation [20,24,41] and the redundancy of Dot1 with other pathways of silencing has made it difficult to elucidate how Dot1 affects chromatin structure and function. In this study, we determined the direct effects of Dot1 and H3K79 methylation by targeting Dot1 to defined places in the yeast genome. Although such targeting can be considered as creating an artificial site of action, this approach has been used previously to identify activities associated directly with the targeted protein and to clarify which of these activities are lost by mutant forms. This approach has been crucial for studying histone modifiers, and provided the first line of evidence that Set2, a histone methyltransferase associated with transcription, has repressive effects on chromatin [42]. Although Dot1 did not act as a transcriptional activator, it antagonized gene silencing, and could do this from a distance. In our search for the underlying mechanism, we identified a methylation-dependent mechanism that affected Sir protein targeting and a methylation-independent mechanism that involved chromatin relocalization and the histone acetyltransferase Gcn5. Our findings show that Dot1 bound to chromatin can positively affect gene expression in a genomic region, partly by inducing chromatin rearrangements over a considerable distance.

## Results

### Dot1 is a derepressor

**Figure 1:**
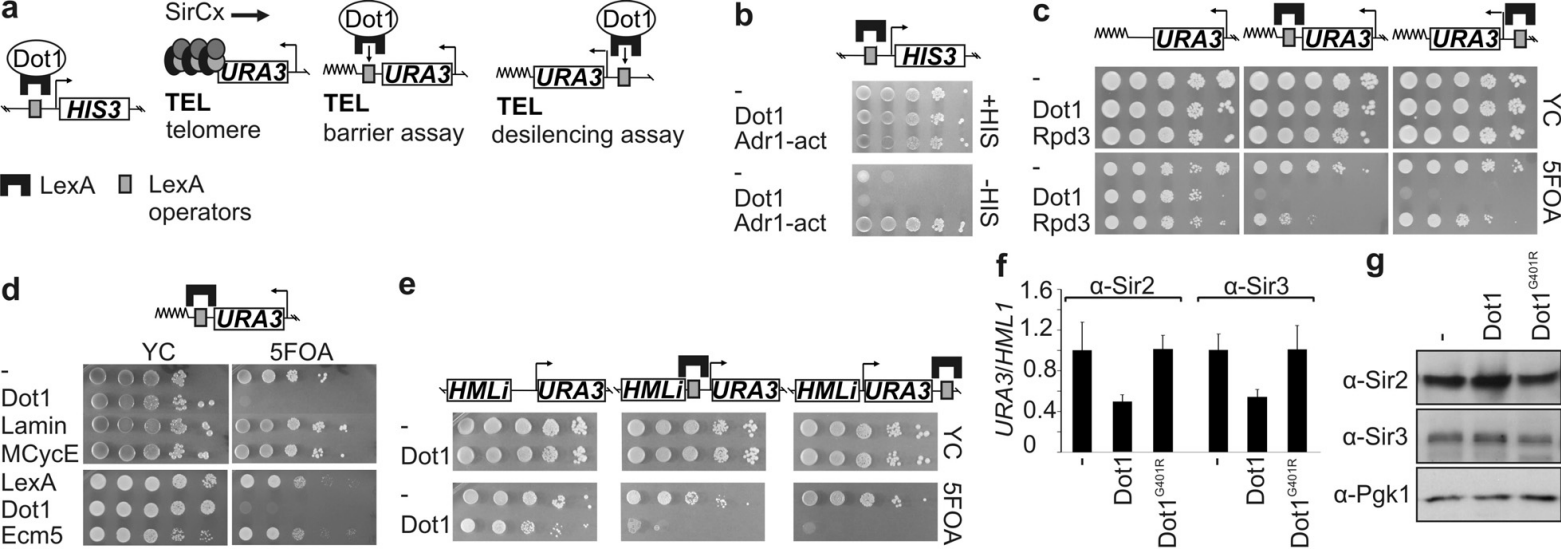
**Dot1 is a derepressor**. **(A) **Targeting of LexA or LexA fusion proteins to LexA operators proximal to a promoterless *HIS3 *gene or a telomeric *URA3 *gene. A telomeric *URA3 *reporter is silenced by the silencing complex (SirCx) that spreads from the telomeric repeats (TEL). LexA-Dot1 was targeted to LexA operators between the telomeric repeats and *URA3 *in a barrier assay, and to LexA operators distal of the telomeric repeats and *URA3 *in a desilencing assay. **(B) **Cells were plated in 10-fold serial dilutions on selective media with or without histidine. Transcriptional activation of *HIS3 *leads to growth on media lacking histidine (strain L40). LexA alone is indicated with a dash. The transcriptional activator domain of Adr1 was used as a positive control. **(C) **Barrier and desilencing assays of Dot1 and Rpd3 targeted to telomere VIIL (strains NKI5128 and NKI5376). A strain without LexA operators (NKI5240) and LexA alone were used as controls. Cells were plated in 10-fold serial dilutions on selective media (yeast culture; YC) with or without 5FOA. Cells that silence *URA3 *can grow on 5FOA media whereas cells that express *URA3 *cannot. **(D) ***URA3 *silencing was not disrupted upon targeting of human Lamin C (pLexA-Lamin), a mutant form of human CyclinE (R130A; pLexA-MCycE) or yeast Ecm5 (pLexA-Ecm5; NKI5128). **(E) **Barrier and desilencing assay of Dot1 at the *HML*α mating-type locus with an inverted I-silencer (*HMLi*; YQY10, YQY09). A strain without LexA operators was used as a negative control (YXB85-n). **(F) **Chromatin immunoprecipitation (ChIP) using specific antibodies against Sir2 and Sir3 [24] was followed by quantitative PCR to determine binding to telomeric *URA3 *and *HML*α upon targeting of Dot1 or Dot1^G401R ^(NKI5128). Average ChIP signals were normalized to input levels and Sir protein binding at *URA3 *relative to *HML*α was plotted (n = 2, +/- SE <). Similar results were obtained with an active (*ACT1*) reference gene (see Additional File 1). **(G) **Immunoblot analysis of Sir2 and Sir3 protein levels in a strain expressing LexA, LexA-Dot1 or LexA-Dot1^G401R ^(NKI5128). Pgk1 was used as loading control.

The effect of local Dot1 binding and activity on gene expression was investigated by fusing *Saccharomyces cerevisiae *Dot1 to the *Escherichia coli *LexA protein and targeting it to LexA operators (LexO), which were engineered into euchromatic and heterochromatic regions of the yeast genome (Figure 1A). In contrast to the known transcriptional activator domain of Adr1, Dot1 and LexA alone did not activate transcription of a promoterless euchromatic *HIS3 *gene (Figure 1B). Therefore, Dot1 did not act as a transcriptional activator.

Next, Dot1 was targeted to heterochromatin, a place where Dot1 is normally not bound [6,7]. In yeast, heterochromatin initiates at silencers of the silent mating-type loci *HML*α and *HMR*a, and at the telomeric repeats, and can subsequently spread along the chromosome [20] in a discontinuous manner [43]. A telomeric *URA3 *gene was used to report changes in chromatin structure by a growth assay [44], allowing rapid screening for chromatin alterations. To examine whether Dot1 can block the spread of heterochromatin as a barrier, a LexA-Dot1 fusion protein was expressed in a strain with LexA operators in between a *URA3 *reporter and the telomeric repeats (Figure 1A). In strains expressing LexA alone or in strains lacking LexA operators, silencing of *URA3 *was unaffected (Figure 1C). However, targeting of Dot1 next to the telomeric repeats disrupted silencing at telomere VIIL (Figure 1C). Thus, Dot1 acted as a barrier at telomeres, which confirms previous findings that targeted Dot1 can prevent ectopic spreading of heterochromatin at a modified *HMR *locus [45]. Rpd3, which was recently identified as a barrier protein [46-48], also acted as a barrier (Figure 1C), whereas targeting of several control proteins did not disrupt *URA3 *silencing (Figure 1D).

Because the effect of Dot1 in the barrier assay was stronger than that of the known barrier protein Rpd3, we examined possible additional effects of Dot1 in a so-called 'desilencing' assay. For this purpose, LexA operators were introduced at the centromeric (distal) side of the telomeric *URA3 *reporter (Figure 1A). Dot1 targeted to the distal LexA operators disrupted silencing, whereas targeted Rpd3 had no or small effects (Figure 1C). Proteins that antagonize local heterochromatin formation from a distance, but do not act as transcriptional activators of non-silenced genes are also referred to as desilencing proteins [49]. Our results show that Dot1 counteracted *URA3 *silencing both in a barrier and in a desilencing assay. We do not know whether barrier-forming and desilencing functions of Dot1 stem from the same activity. Based on these findings, we henceforth refer to Dot1 as a derepressor.

To further investigate the derepressor activity of Dot1 and to exclude possible telomere-looping effects [50], Dot1 was targeted to a mating-type locus internal to the chromosome, *HML*α, in which the I-silencer element was inverted to allow spreading of the Sir complex to the neighboring *URA3 *reporter (Figure 1E) [51]. In addition, at this non-telomeric native locus, LexA-Dot1 disrupted silencing both in a barrier and a desilencing assay and, importantly, did not affect *URA3 *silencing in a strain without LexA operators (Figure 1E). Because the distal LexA operators and the *URA3 *promoter are 1.7 kb apart from each other, Dot1 displayed derepressor activity from a distance (Figure 1E). The changes that we observed in the quantitative growth assays (and *URA3 *expression by reverse transcriptase quantitative (RT-q)PCR, see below) indicated that changes had occurred in the silent chromatin domain around the *URA3 *reporter gene.

We next investigated the mechanisms by which bound Dot1 can affect a silenced domain. Chromatin immunoprecipitation (ChIP) analysis showed that upon Dot1 targeting, Sir2 and Sir3 protein binding to telomeric *URA3 *was reduced by two to three times (Figure 1F; see Additional file 1) whereas global Sir2 and Sir3 expression was unaltered (Figure 1G). Therefore, at least part of the loss of silencing mediated by Dot1 seems to originate from a decrease in Sir protein binding. Targeting of a catalytically inactive Dot1^G401R ^protein [2] did not affect Sir protein binding (Figure 1F). We conclude that Dot1-mediated methylation directly affects Sir mediated chromatin and silencing from a distance, by antagonizing Sir complex binding.

### Dot1 displays a methyltransferase-dependent and -independent derepressor activity

**Figure 2:**
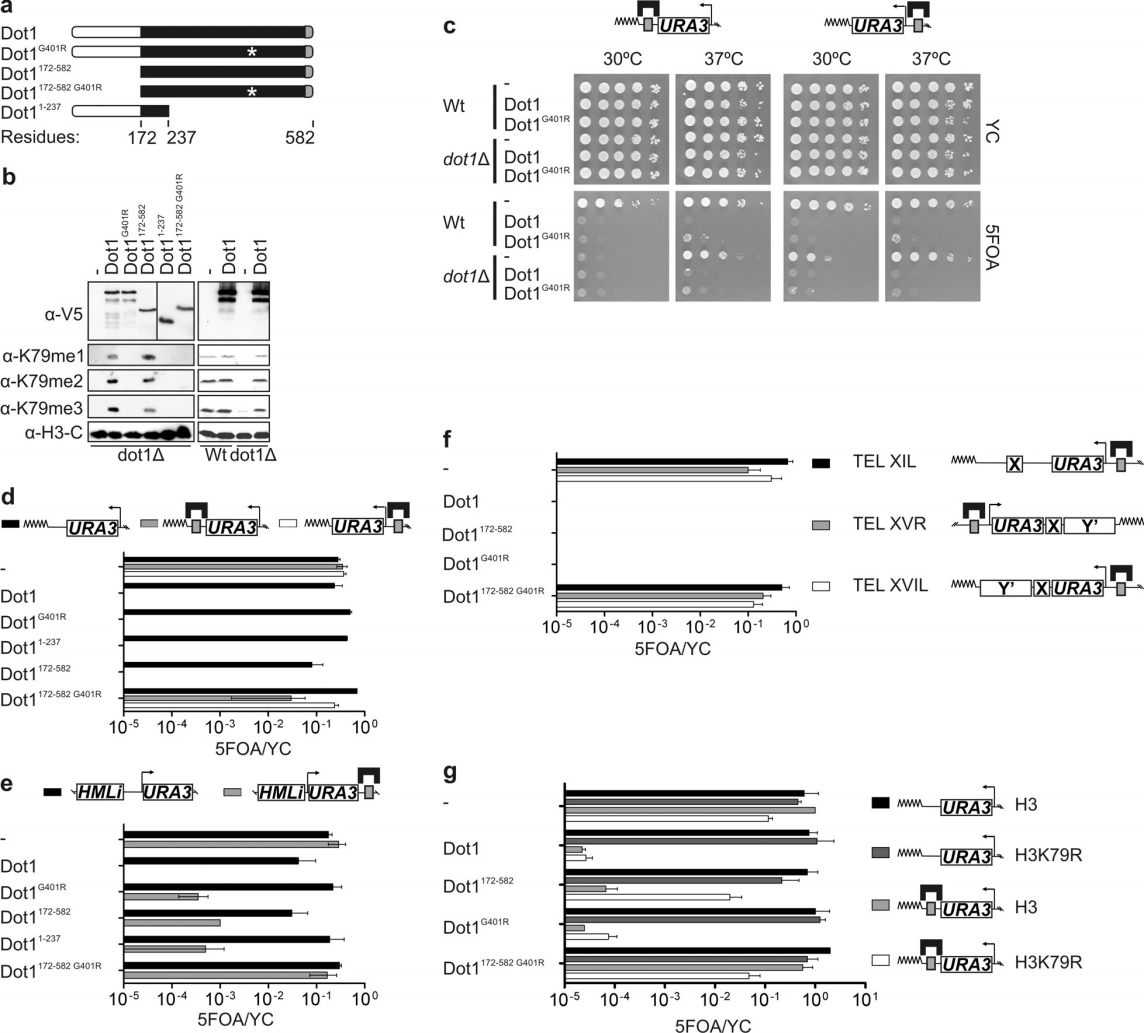
**Dot1 is a methyltransferase-dependent and -independent derepressor**. **(A) **Outline of Dot1 deletion mutants showing the N terminus (white), the methyltransferase domain (black) and the H4 binding domain (grey). The G401R mutation (*) abolishes the catalytic activity of Dot1. All fusion proteins contained LexA and a V5 tag at the N terminus. **(B) **Protein and H3K79 methylation levels of Dot1 mutants described in (A) were determined in a *dot1*Δ strain lacking LexA operators (NKI5070). H3K79 methylation levels were determined of a wild-type strain or a *dot1Δ *strain (NKI5376 and NKI5378) expressing LexA or LexA-Dot1. Protein expression and H3K79 methylation were determined by immunoblot analysis using a V5 antibody and antibodies specific for H3K79 mono-, di- and trimethylation or the histone H3 C terminus. **(C) **Barrier (NKI5128 and NKI5129; left) and desilencing assay (NKI5376 and NKI5378; right) in the presence and absence of *DOT1*. Deletion of *DOT1 *results in reduced silencing that could be bypassed at 37°C [24,25]. Both LexA-Dot1 and LexA-Dot1^G401R ^were still able to disrupt *URA3 *silencing in a *dot1*Δ strain at 37°C, showing that derepressor activity does not require involvement of endogenous Dot1. Note that *URA3 *silencing is not completely lost in *dot1*Δ at 30°C (NK5378). This is caused by enhancement of telomeric silencing by the *TRP1 *gene distal to *URA3 *(see Additional file 2A). **(D) **Derepressor activity of Dot1^G401R ^and Dot1 deletion mutants at telomere VIIL (strains NKI5240, NKI5128 and NKI5376). Serial dilutions as presented before were quantified and plotted as bar graphs. **(E) **Derepressor activity of Dot1^G401R ^and Dot1 deletion mutants at *HML*α (strains YXB85-n and YQY09). Growth on 5FOA observed for LexA-Dot1^G401R^, LexA-Dot1^172-582 ^and LexA-Dot1^1-237 ^when targeted to LexA operators was caused by colonies that were uracil auxotrophs, which most likely represent *URA3 *mutants. **(F) **Derepressor activity of Dot1 and Dot1 mutants at the native chromosomes XIL, XVR and XVIL as described previously [82] (NKI2229, NKI2230 and NKI2231). **(G) **Dot1 and Dot1 mutants were expressed in *dot1Δ *strains with or without LexA operators, expressing wild-type histone H3 or histone H3 with K79 mutated to arginine (H3K79R), to determine whether K79 methylation is required for the methyltransferase-dependent derepressor activity (NKI6045, NKI6047, NKI6049 and NKI6051). Strains were grown at 37°C to enhance *URA3 *silencing.

To further determine the mechanism of chromatin derepression, deletion mutants of Dot1 were generated (Figure 2A). The N-terminal domain (Dot1^1-237^) has been implicated in chromatin binding [52]. The C-terminal domain (Dot1^172-582^) harbors the catalytic activity and the acidic patch that binds to the basic patch on the tail of histone H4 [26,27,52]. LexA-Dot1 fusion proteins with an intact catalytic domain restored global H3K79 methylation, whereas inactive Dot1 fusions did not complement the *dot1Δ *(Figure 2B).

Unexpectedly, although targeting of the catalytically inactive Dot1^G401R ^did not alter Sir protein binding at the telomere (Figure 1F), Dot1^G401R ^did show derepressor activity (Figure 2CD). This effect was not due to protein overexpression alone, but acted in *cis*, because Dot1^G401R ^did not affect silencing of a reporter lacking adjacent LexA operators (Figure 2D). To exclude the possibility that the Dot1^G401R ^protein formed a heterodimer with the catalytically active endogenous Dot1, we also targeted Dot1^G401R ^in a *dot1*Δ strain. Because deletion of *DOT1 *results in reduced silencing, the barrier and desilencing assays were performed at 30 and 37°C, because high temperature enhances silencing and bypasses the need for endogenous Dot1 to silence *URA3 *[24,25]. Both LexA-Dot1 and LexA-Dot1^G401R ^were still able to disrupt *URA3 *silencing in a *dot1*Δ strain at 37°C, showing that derepressor activity does not require involvement of endogenous Dot1 (Figure 2C).

To map the derepressor domain, we analyzed the Dot1 deletion mutants. Interestingly, both the N-terminal domain (Dot1^1-237^) and the methyltransferase domain (Dot1^172-582^) functioned as derepressors in both the barrier assay and the desilencing assay at telomeres and the *HML*α locus (Figure 2D-E). However, a catalytically inactive methyltransferase domain (Dot1^172-582 G401R^) did not disrupt silencing (Figure 2D-E). Different native telomeres and truncated telomeres can show different silencing properties, Sir protein binding, and nucleosome positioning [29,53,54]. To test whether the derepressor activity of Dot1 is a general property or is restricted to the truncated telomere used here, LexA operators and the *URA3 *gene were introduced at three different native chromosome ends. Dot1 derepression activity was very similar at truncated telomeres and at native telomeres with or without a subtelomeric 'Y' element (Figure 2F).

The only known substrate of Dot1 is histone H3K79. To verify whether the methyltransferase-dependent pathway was mediated by methylation of H3K79, we replaced histone H3 by a H3K79R mutant and grew strains at 37°C to partially suppress the *URA3 *silencing defect in this mutant (Figure 2G) [55]. Targeting of LexA-Dot1^172-582 ^resulted in derepressor activity in the presence of histone H3 but not in the presence of H3K79R (Figure 2G). Together, our findings show that Dot1 harbors two redundant derepressor mechanisms: one methyltransferase-independent mechanism mediated by the N terminus, and one mechanism mediated by its methyltransferase activity towards H3K79.

### Gcn5 is required for the methyltransferase-independent derepression by Dot1

**Figure 3:**
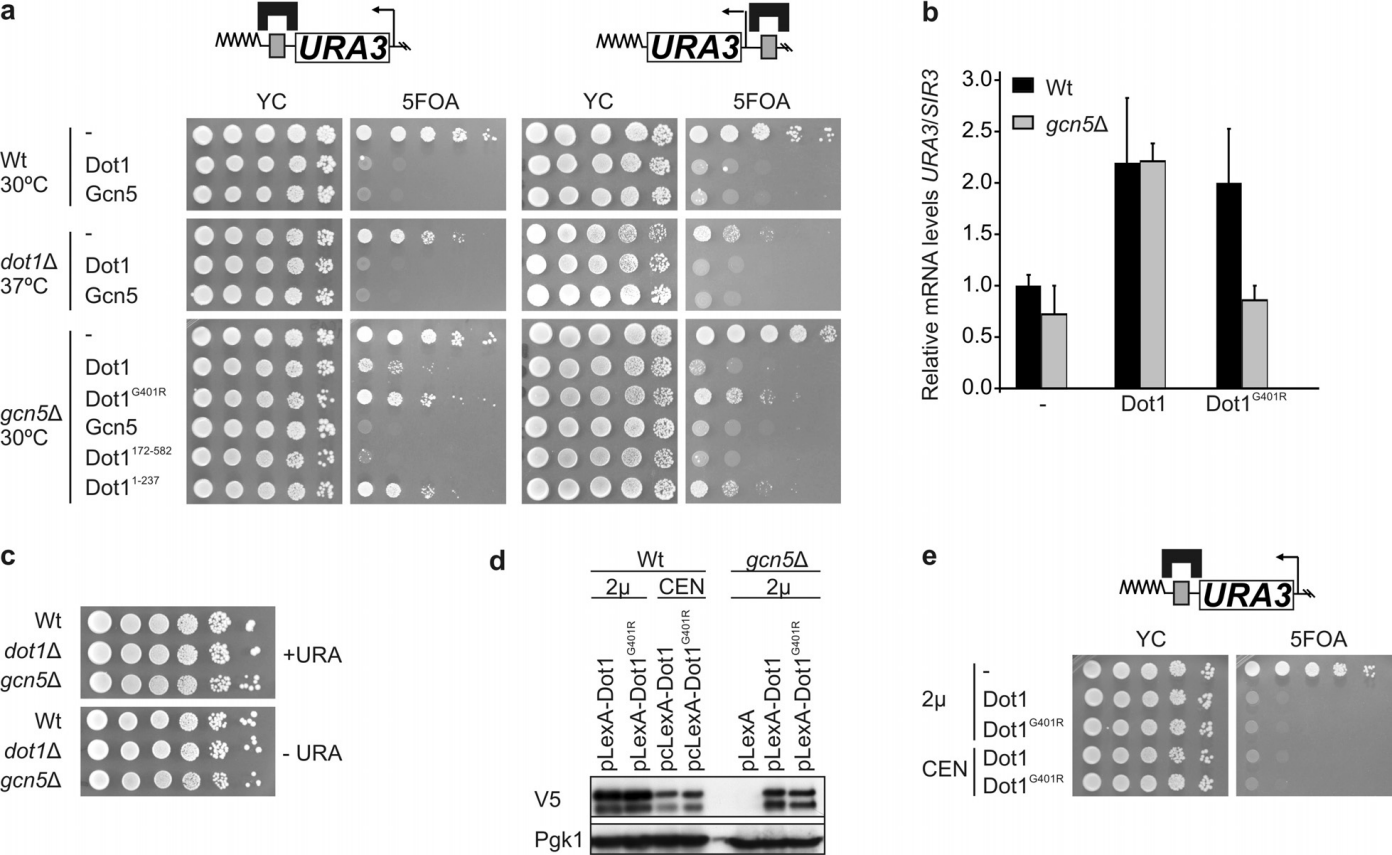
**Derepression by Dot1 requires the histone acetyltransferase Gcn5**. **(A) **Derepressor activity of Dot1 and Gcn5 in a wild-type strain (NKI5128 and NKI1088), a *dot1*Δ strain (NKI5129 and NKI6020) or a *gcn5*Δ strain (NKI5399 and NKI6018). Strains lacking endogenous Dot1 were grown at 37°C to suppress the silencing defect, (see Figure 2C). We found that Gcn5 had a more prominent role in the barrier than in the desilencing assay of Dot1. *URA3 *silencing in the *dot1Δ *strain at 37°C in the desilencing assay (NKI1088 background) was limited compared with *URA3 *silencing in the *dot1Δ *at 37°C in the barrier assay (NKI1084 background), which is the result of the telomeric context (see Additional file 2A). **(B) ***URA3 *expression (n = 2 +/- SEM) was determined by reverse transcriptase-PCR and normalized to *SIR3 *expression, which is expressed at similar levels as *URA3 *and at equal levels in wild-type cells and histone modifier mutants [24]. **(C) **WT, *dot1*Δ and *gcn5*Δ strains with a *URA3 *gene at its endogenous euchromatic location were grown on media with or without uracil (BY4702, NKI3006 and NKI1107). Growth on media lacking uracil requires activation of *URA3 *by Ppr1, which is not affected by the loss of Dot1 or Gcn5. **(D) **LexA, LexA-Dot1 and LexA-Dot1^G401R ^were expressed from a high copy 2 μ plasmid (used for all experiments described here) in a wild-type strain (*GCN5*; NKI5128) or a *gcn5*Δ strain (NKI5399), and compared with wild type strains expressing LexA fusion proteins from a single-copy CEN (centromere sequences) plasmid. Each LexA-tagged protein also contained a V5 tag, which was used for immunoblot detection. Pgk1 was used as loading control. **(E) **Barrier assay of the LexA-tagged proteins expressed from the plasmids described in (D).

To further investigate the mechanisms of methylation-dependent and -independent derepression by Dot1, we examined the role of Gcn5. This histone acetyltransferase also displayed derepressor activity (Figure 3A), as has been reported previously [46,49,56-58]. To test whether Gcn5 and Dot1 are functionally related, their derepression activities were measured in strains lacking either endogenous Dot1 or Gcn5. Derepression by Gcn5 did not require Dot1 (Figure 3A). However, methyltransferase-independent derepression by Dot1^G401R ^and Dot1^1-237 ^was partially compromised in strains lacking Gcn5 (Figure 3A). Furthermore, LexA-Dot1^G401R ^did not increase *URA3 *mRNA levels in *gcn5*Δ cells, whereas catalytically active Dot1 proteins still showed strong derepression (Figure 3B). This confirms the anti-silencing character of H3K79 methylation. Together, our results show that the methyltransferase-independent derepressor activity mediated by the N-terminal domain of Dot1 requires the presence of Gcn5. The role of Gcn5 is most probably mediated by its catalytic activity (see Additional file 2B), but Rsc4, a substrate of Gcn5 [59], was not required (see Additional file 2C). We found that loss of Gcn5 did not affect normal activation of *URA3 *(Figure 3C), but did lead to reduced expression of the LexA-Dot1 fusion proteins (Figure 3D). However, in the presence of Gcn5, low expression levels of LexA-Dot1 were sufficient for derepression, showing that the loss of Dot1-mediated derepression in the *gcn5Δ *strain was not caused by reduced expression of the LexA fusion proteins (Figure 3E). Finally, analysis of several other histone modifiers associated with active chromatin showed that the role in Dot1 derepressor activity is specific for Gcn5 (see Additional file 2(D, E)). Because the loss of *URA3 *derepression is not complete in a *gcn5Δ *strain, these experiments also indicated that another pathway is involved in depression via the N-terminal domain of Dot1.

### Tethered Dot1 caused chromatin relocalization

**Figure 4:**
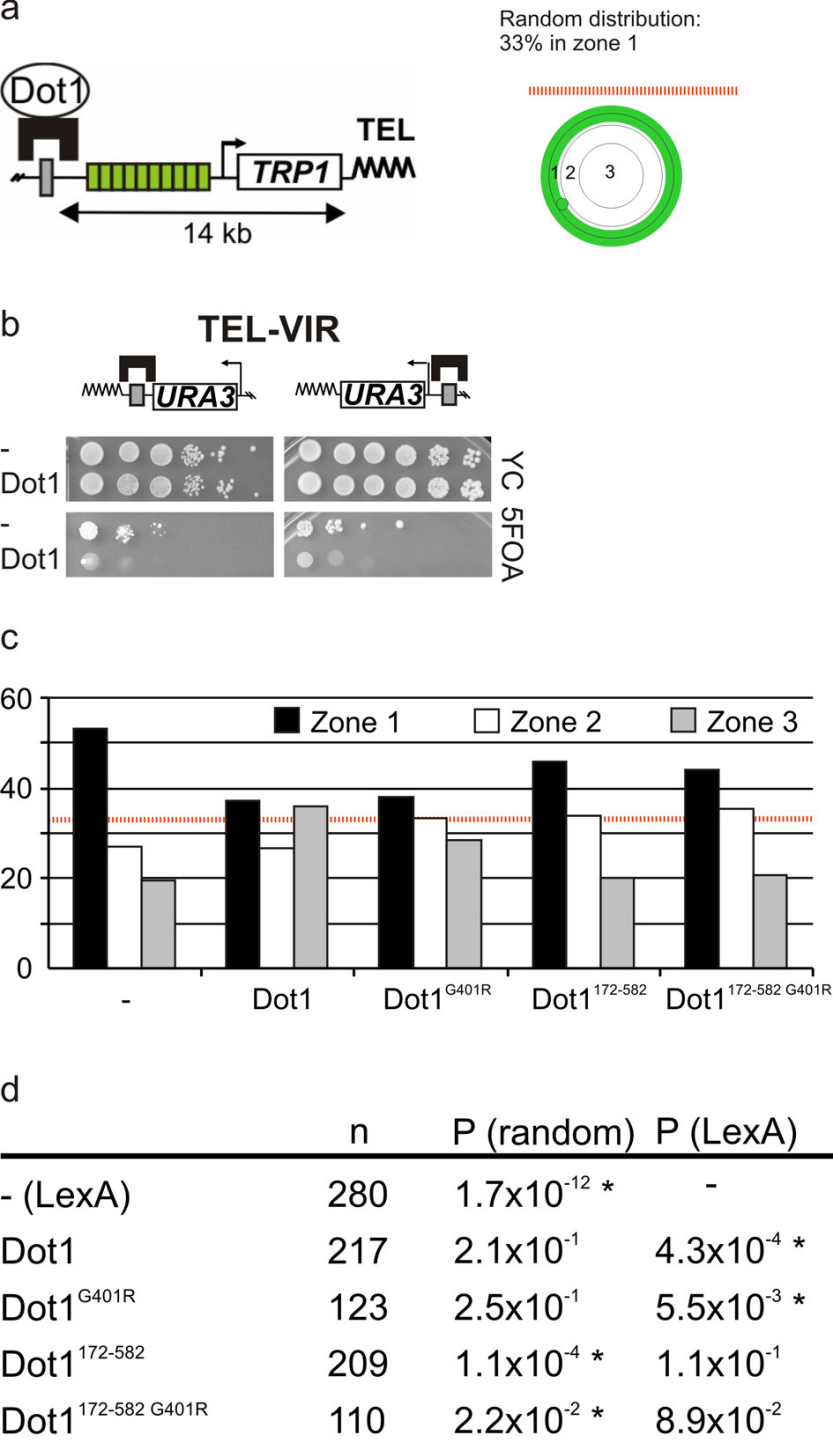
**Tethered Dot1 disrupts telomere anchoring**. **(A) **Telomere anchoring was measured in strain GA-1459. TEL VIR was visualized by binding of a GFP-LacI fusion protein to the Lac operators (indicated by green boxes). Subnuclear position was scored relative to the nuclear envelope tagged by a GFP-Nup49 fusion in approximately 100 to 300 nuclei. **(B) **Derepressor activity of targeted Dot1 at TEL VIR was measured in strains NKI1117 and NKI1118. **(C) **Localization data are represented in bar graphs as the percentage of spots in one of three concentric zones of equal surface. The dashed line at 33% corresponds to a random distribution. Spots observed in zone 1 represent telomeres localized to the nuclear periphery. **(D) **Two different statistical tests were performed. First, we tested whether telomeres targeted with LexA fusion proteins showed a random distribution over the three zones in the cell. Second, whether telomeres targeted with LexA-fusion proteins had a similar distribution to that of telomeres targeted with LexA alone. A significant difference for each Dot1 protein could be identified in at least one of the two tests. Asterisk indicates statistically significant differences (*P *< 0.05) from random telomere distribution (*P*_(random)_) or from telomere distribution upon LexA targeting (*P*_(LexA)_). The number of cells analyzed is indicated by *n*.

To gain more insight into the possible methylation-independent mechanisms through which Dot1 derepresses Sir-repressed genes, we investigated whether Dot1 binding affected anchoring of the silenced telomere to the nuclear periphery. In yeast and metazoans, heterochromatin often clusters next to the nuclear envelope [60-63]. This clustering and peripheral localization contributes to heterochromatin establishment and maintenance [64]. We monitored the localization of a native telomere in living cells by microscopy, using a strain that has telomere VIR tagged with an array of Lac operators bound by green fluorescent protein (GFP)-LacI and telomeric LexA operators (Figure 4A). In a different strain, we confirmed that targeting of Dot1 caused derepression of a proximal *URA3 *reporter gene at telomere VIR (Figure 4B). The GFP-LacI labeled telomere bound by LexA was predominantly perinuclear (zone 1 as scored by position relative to the nuclear periphery), whereas a telomere bound by wild-type Dot1 had a significantly different localization, being more randomly distributed in the nucleus (Figure 4CD). Therefore, the derepression by Dot1 was accompanied by loss of perinuclear localization of the heterochromatic region.

Similar to its effect on chromatin derepression (Figure 2), the methyltransferase inactive LexA-Dot1^G401R ^mutant also provoked the relocalization of telomere VIR away from the nuclear envelope (Figure 4CD). By contrast, binding of a catalytically active or inactive Dot1 protein lacking the N terminus (Dot1^172-582 ^and Dot1^172-582 G401R^) maintained significant perinuclear enrichment, similar to that observed in the presence of LexA alone (Figure 4CD). These results show that chromatin-bound Dot1 can move a telomere away from the periphery and, remarkably, that the methyltransferase activity of Dot1 is neither necessary nor sufficient for this effect. Rather, the N-terminal domain is required. Analysis of the role of Gcn5 in Dot1-mediated telomere relocalization is complicated by growth defects caused by deletion of Gcn5 in the strain background used. However, by analyzing a different native chromosomal locus, we found that Gcn5 could indeed promote relocalization of a chromatin domain from the nuclear periphery to the nuclear interior (see Additional file 3). These results indicate that chromatin localization away from the nuclear periphery by the N terminus of Dot1 may be mediated by Gcn5.

## Discussion

Dot1-mediated H3K79 methylation has been correlated with both increased and decreased transcription in different organisms, yet it has been difficult to distinguish global or indirect effects from local effects when interpreting *dot1Δ *loss-of-function mutations. This is even more difficult in budding yeast, where the role of Dot1 in gene silencing appears to be masked by redundant pathways [24]. Nonetheless, in our study we were able to circumvent indirect effects by targeting Dot1 to defined sites in the yeast genome and comparing the resulting phenotypes with those of a strain that lacks the target site. We found that Dot1 binding does not activate transcription in yeast, yet it antagonizes heterochromatin-mediated gene silencing. Furthermore, we found that Dot1 does not simply act as a barrier to block the spreading of heterochromatin, but actively derepresses silent chromatin from a distal position. Because Dot1 behaved similarly in both the barrier and desilencing assays, it seems likely that it acts in both assays through a common mechanism. Moreover, the ability to derepress was not restricted to yeast Dot1, because similar effects were obtained for human DOT1L when it was targeted to a yeast telomere (see Additional File 4).

Separation-of-function alleles of Dot1 show that Dot1 derepresses by two redundant mechanisms. One mechanism of derepression is mediated by H3K79 methylation, which has been shown *in vitro *to reduce the affinity of chromatin for Sir3 [25,27,32,33,35]. Our results provide direct evidence for competition between H3K79 methylation and Sir protein binding *in vivo*. It remains unclear, however, whether targeted Dot1 is sufficient to derepress *URA3*, or whether it also requires additional (non-targeted) Dot1 molecules activated by elongating RNA polymerase. It is possible that initial transcription events lead to the recruitment of the Paf1 elongation complex and subsequent ubiquitylation of H2B. Both these processes would in turn stimulate the methylation of H3K79 by Dot1 [6,65-70].

The second derepression mechanism, which does not affect Sir protein binding, requires the histone H3 and H4 histone acetyltransferase Gcn5. How might Gcn5 assist Dot1? The genetic requirement for Gcn5 suggests that the Dot1 N terminus may recruit Gcn5 to sites at which Dot1 is bound. However, we could not detect a direct interaction between the two proteins by ChIP, yeast two-hybrid, co-immunoprecipitation or *in vitro *pull-down experiments with recombinant proteins. An alternative possibility is that Gcn5 triggers derepression through its global non-targeted histone acetyltransferase activity [71,72]. One plausible scenario is that the targeted N terminus of Dot1 alters local chromatin structure, allowing Gcn5 to acetylate histone tails to which it might otherwise not have access because of the local heterochromatic structure. Intriguingly, the interaction between Dot1 and the basic patch on the histone H4 tail (which is also bound by Sir3 [26,27]) is not sufficient to trigger methyltransferase-independent derepression, as LexA-Dot1^172-580 G401R^, which lacks the N terminus but still contains the C-terminal H4-binding domain, failed to derepress (Figure 2). Indeed, we found that the N terminus alone is sufficient for Dot1-mediated derepression (Figure 2).

Several other factors have been identified that can act as anti-silencers, although how they affect gene expression at a distance is unclear [49]. We explored potential mechanisms through which Dot1 might derepress silencing at a distance. In yeast, transcriptional regulators typically do not function from downstream positions, but act by binding to upstream activation sequences (UAS) that are positioned within a few hundred base pairs from the transcription start site [50,73]. This is in contrast to gene regulation by enhancers (the UAS counterparts in mammalian cells), which can act over longer ranges and also from downstream positions [74]. Silencing in yeast is highly sensitive to local Sir factor concentration, and is therefore strongly enhanced by the recruitment of silencers and silent domains to the nuclear envelope [75], where Sir proteins accumulate in foci that colocalize with clusters of yeast telomeres. Quantitative analysis of subnuclear localization induced by Dot1 targeting suggests that Dot1 contributes to derepression by promoting the relocation of a domain away from the repressive environment at the nuclear periphery.

In budding yeast, the telomeres and the two silent mating-type loci, *HML *and *HMR*, co-localize in four to eight clusters at the nuclear periphery. Many Sir binding sites are created by the binding of Rap1 to the telomeric repeats and the anchoring of telomeres through both yKu and Sir4, leading to the sequestration of Sir proteins away from the rest of the genome [64,76]. Positioning a promoter or gene near the telomere (or indeed anywhere at the nuclear envelope) in a strain that has functional telomere anchoring facilitates stable gene repression, as long as the targeted reporter contains Sir nucleating elements, such as silencers or protosilencers [61,77-79]. The relocation of a region bound by Dot1 is likely to influence the subnuclear position of neighboring genes because chromatin is a contiguous compacted fiber with limited flexibility, and this would explain why tethered Dot1 functions as derepressor from a distance.

Loss of anchoring was achieved by targeting of the activity-deficient Dot1^G401R^, suggesting that the methyltransferase-independent derepression described above depends on or correlates with relocalization (Figure 4). Interestingly, conditions that lead to telomere relocalization (targeting of Dot1^G401R^) did not lead to detectable changes in Sir protein binding (Figure 1F). Whether targeting of a catalytically inactive Dot1 caused qualitative changes in chromatin or small quantitative changes that remained undetected by ChIP is still unknown.

Telomeric heterochromatin is anchored to the nuclear periphery by two pathways, one of which is enhanced by formation of silent chromatin, whereas the other involves the DNA-end binding complex yKu [61]. Although the local H3K79 methylation mediated by targeted Dot1 was able to disrupt Sir complex association [27,32,33], it was not sufficient to cause loss of telomere anchoring (Figure 1, 2, 4). This is most likely due to a redundant anchoring pathway mediated by yKu, which helps tether telomeres in the absence of Sir-mediated repression [80].

The distal effects of Dot1-mediated H3K79 methylation on gene silencing are in line with observations that yeast heterochromatin spreads in a discontinuous fashion [43]. The establishment of silent chromatin domains at yeast telomeres is determined by anti-silencing and relay elements [29,54,81,82]. Therefore, one possibility is that Dot1 bound at distal sites also disrupts the interaction between such relay elements. We found that a nourseothricin resistance (NatMX) gene cassette was also able to disrupt gene silencing from a distal position (see Additional file 2A), lending further support for the notion that distal elements can influence silencing at telomere proximal positions.

## Conclusions

Previous analyses showed that histone H3K79 methylation by Dot1 in euchromatin indirectly promotes heterochromatin formation. In this paper, we show that at a local level Dot1 can counteract heterochromatin formation by H3K79 methylation and chromatin relocalization, which is in agreement with the observed loss of silencing by very high overexpression of Dot1 [2,21,27,83]. Together, these results suggest that Dot1 functions as complex modulator of heterochromatin organization. The relative strength of the indirect and more direct Dot1 activities, gene-specific characteristics, and the contribution of other pathways of heterochromatin formation will together fine-tune heterochromatin establishment and gene expression.

Human DOT1L also derepressed silent chromatin in yeast (see Additional file 4). If human DOT1L plays similar roles in mammals, mistargeting of DOT1L in human leukemia might antagonize gene silencing or repression, and thereby lead to higher gene expression levels. Although the catalytic activity of DOT1L has been shown to be required for leukemic transformation and maintenance of the transformed state by MLL and CALM fusion proteins in *in vitro *models, it is possible that DOT1L collaborates with other euchromatic modifiers such as Gcn5 to establish and maintain the altered gene expression levels in human leukemias. Identification of the molecular mechanisms of derepression by human DOT1L will be crucial for understanding the role of DOT1L in leukemias bearing MLL and CALM fusion proteins.

## Methods

### Yeast strains and plasmids

The yeast strains and plasmids used in this study are specified in the supplementary material (see Additional file 5 Additional file 6. To obtain strains with a *URA3 *reporter for telomeric silencing, telomere VIIL was truncated at *ADH4 *by integration of URA3-LexO_3_-TEL-VIIL (three LexA binding sites; pVIIL-URA3-LexAS3-TEL [84]), URA3-LexO_2_-TEL-VIIL (two LexA binding sites; pVIIL-URA3-LexAS2-TEL [84]), URA3-TEL-VIIL (pADH4UCA-IV [85]) or LexO_10_-URA3-TEL-VIIL (pT7 [86]) in an Y7092 background (NKI1084, NKI5070, NKI5072, NKI5128, NKI5240, NKI5376, NKI5420, NKI5422). A NatMX selection cassette was introduced by homologous recombination distal to the LexA operators in NKI5376 (NKI1088; primers ADH4tINT1KO and ADH4tINT2KO). Strain NKI1117 and NKI1118 were generated by targeting the URA3-LexO_3_-TEL barrier cassette from pVIIL-URA3-LexO_3_-TEL or the TRP1-LexO_10_-URA3-TEL desilencing cassette from pT7, respectively, to telomere VIR in strain BY4733, thereby truncating the endogenous telomere. The cassettes were amplified using primers URA3_TELVIRtr_F1 and URA3_TELVIRtr_F2, respectively and URA3_TELVIRtr_R1. A genomic region of TEL VIR was amplified using primers TELVIR-3907_F and TELVIR-3463_R, and subsequently fused to the reporter cassettes by overlap PCR. Primers used to target TEL VIR are shown in the primer list. A LexO_5_-URA3 desilencing cassette was targeted to native telomeres of TEL XIL (NKI2229), TEL XVIL (NKI2230) and TEL XVR (NKI2231) of strain Y7092. A LexO_5 _region was amplified from plasmid pT7 using primers LUJ2 and DLU-11L-DF2 or DLU-15R/16L-DF2. The URA3 gene was amplified from pURA3-TEL VIIL using primers LUJ1 and BDUL-11L-R1 or BDUL15R/16L-R1. The fragments were fused by overlap PCR and targeted to position P2 (11L) or P1 (15R/16L), as described previously [82]. Integrations were verified by PCR using telomere-specific primers and by sequencing of regions around the insertion site. Strains NKI1043 and NKI6041-NKI6051 were derived from UCC1369 [2]. UCC1369 was crossed with BY4727 to remove silencing reporters and hhf1-hht1Δ::LEU2 was replaced by hhft1-hht1Δ::HIS3 to obtain NKI6041. URA3-LexO_3_-TEL VIIL or URA3-TEL VIIL was targeted to telomere VIIL of NKI6041 to obtain NKI6042 or NKI6043, respectively. Subsequently, pMP9 was replaced by pMP3 (H3, NKI6045 and NKI6047) or by pFvl88 (H3K79R, NKI6047 and NKI6051) via a plasmid-shuffle procedure. Strains harboring a gene-specific knockout were made using the plasmids pRS400, pFvl99 and pFvl100.

Plasmid pFvL232 was made by amplification of the P_ADH1_-LexA fragment from pBTM116 using a three-step PCR protocol (primers LexADot1V5P2 and LexAV5P3) that resulted in the introduction of a V5 tag (GKPIPNPLLGLDST). The appended *Not*I and *Spe*I sites were used to clone the fragment into pRS425. Plasmid pFvL230 was made by performing several steps. First, the full-length Dot1 open reading frame (ORF) was amplified with primers that included a 5' *Eco*RI site and 3' *Bam*HI site to clone the Dot1 ORF into pBTM116 and generate pFvL23. Using a three-step PCR protocol, a V5 tag was inserted in between LexA and Dot1 to generate P_ADH1_-LexA-V5-Dot1 (primers LexADot1V5P2 and LexADot1V5P3), and the resulting PCR fragment was cloned in pRS425 using the appended *Not*I and *Spe*I sites. pFvL909 was generated by gap repair of pFvL230 using double-strand oligonucleotides to introduce an SV40 nuclear-localization signal (NLS) (PKKKRKV) [87] and a unique *Nru*I site. A Dot1^G401R ^mutant (pFvl908) was generated by oligo-mediated site-directed mutagenesis on pFvL230. Deletion mutants of Dot1 (pFvl905, pFvl913, pFvl901) were made by replacing full length Dot1 in pFvL230 with PCR-amplified Dot1 deletion fragments by cloning or by inserting the fragments into pFvL909 by gap repair to include the SV40 NLS. Plasmid pIS001 was made by gap repair using a G401R fragment of DOT1 and plasmid pFvL901 digested with *Bsa*BI and *Nhe*I. Plasmids pFvL914 and pFvL916 were generated by gap repair by co-transformation of the LexA-V5-Dot1 fragment from pFvL230 and pFvL908 digested with *Bsr*GI-XbaI and pRS315 digested with *Alw*NI and *Eco*RI. Deletion of the N-terminal domain disrupted a putative NLS. Although LexA has been suggested to have nuclear-localization properties [88], Dot1 mutants without an N-terminal domain were fused to an NLS. Plasmids pFvL925 and pFvL927 were made by PCR amplification of N-terminal fragments of human Dot1 from pCDNA3B-FLAG-hDot1L and replacement of yeast Dot1 in pFvL230 by gap repair. Plasmid pFvL921 was made by amplification of the RPD3 ORF from genomic DNA and replacement of Dot1 in pFvL230 by gap repair. Plasmid pFvl250 was made from a MORF-ECM5-HA-TAP plasmid (pYMR176) in two steps. First, the *URA3 *marker was replaced with a *LEU2 *marker by gap repair using the *Aat*II/*Pvu*II *LEU2 *fragment of pRS305. Next, the *GAL1 *promoter was replaced by a 1.2 kb fragment containing the *ADH1 *promoter linked to LexA-V5 (from pFvL230) and an upstream hygromycin resistance (HphMX) marker (from pFvL100) was inserted. Plasmids pRS400, pFvL99 and pFvL100 were used for gene replacements by KanMX4 (kanamycin resistance), NatMX4 and HphMX4, respectively. To generate pFvL99 and pFvL100, the *Pac*I/*Bsm*I KanMX4 insert of pRS400 was replaced by the *Pac*I/*Bsm*I insert of pAG25 or pAG32 [89], respectively. The drug-resistance cassettes were amplified using the standard pRS primers [90].

Yeast growth and silencing assays were performed as described [21]. To analyze gene expression of the *URA3 *reporter, strains were plated in 10-fold dilution series on media with or without 5-fluoroorotic acid (5FOA), which is toxic and inhibits growth when *URA3 *is expressed [44]. Media for growth assays of strains expressing a LexA-fusion protein lack leucine to select for presence of the plasmid. Growth assays to analyze *HIS3 *activation were performed on media lacking leucine and histidine.

### Protein analysis

Cell extracts were made as described previously using glass beads and SUMEB buffer containing phenylmethylsulfonyl fluoride (1 mmol/l), benzamidine (5 mmol/l), pepstatin (1.5 mmol/l), leupeptin (2 mmol/l) and dithiothreitol (1 μmol/l) [91]. Primary antibody incubations were performed in Tris-buffered saline-Tween with 2% dry milk. Primary antibodies used for immunodetections were V5 antibody (R960-25; Invitrogen, Breda, The NetherlandsH3, H3K79me1, H3K79me2 and H3K79me3 [21], PGK1 (A-6457; Molecular Probes, Breda, The Netherlands).

### Chromatin Immunoprecipitation

ChIP was performed as described previously [2]. Chromatin was sheared for 6 minutes with 30 second intervals, using a Bioruptor (Diagenode). ChIP analyses were performed with anti-H3 [21], anti-Sir2 (yN-19; Santa-Cruz) and anti-Sir3 [2] antibodies coupled to magnetic beads (Dynabeads; Invitrogen). ChIP DNA was quantified by real-time qPCR analysis using a commercial master mix and thermal cycler (SYBR^® ^Green PCR Master Mix and ABI PRISM 7500; Applied Biosystems). A standard curve was made from one of the input samples, which was then used to calculate the relative expression of the other samples using 7500 Fast System software. For primers, see Additional file 7.

### mRNA quantification

RNA was extracted (RNeasy Kit; Qiagen), and cDNA made by using reverse transcriptase (Super-Script II; Invitrogen). RT-PCR fragments were separated on gels and quantified using the TINA 2.09 software (Raytest). Primers are described in Additional file 7. Gene expression profiling of a *dot1Δ::KanMX *strain derived from BY4742 was performed as described previously [92].

### Live imaging of telomere localization

For live imaging, yeast strain GA-1459 was used, which has TEL VIR tagged with four LexA operators (LexO) and an array of Lac operators (LacO), and expresses GFP-LacI to visualize the LacO and GFP-Nup49 to tag the nuclear periphery [93]. Cultures of GA-1459 containing LexA plasmids were grown in synthetic medium lacking leucine to a concentration of 0.2-0.4 × 10^7 ^cells/ml. For subnuclear position analysis, living cells were imaged at 30°C on agarose patches containing synthetic complete medium + 4% glucose as described [93]. Stacks of 21 images of 0.2 μm step size were captured on a wide field microscope (Metamorph-driven IX70; Olympus) equipped with a camera (Coolsnap HQ; Roper Scientific Photometrics). The radial position of tagged TEL VIR was assigned to one of three concentric zones of equal surface in the focal plane bearing the brightest GFP-LacI focus as described [93]. Nuclei in which the focus was detected in the three top or bottom focal planes were excluded from the analysis. A χ^2 ^test was used to determine if the measured frequency of TEL VIR position in zone 1 differed from a random distribution using a 95% confidence limit. A proportional analysis was used to measure confidence values between two strains. For each strain, nuclei from four to five independent cultures were combined.

## Competing interests

The authors declare that they have no competing interests.

## Authors' contributions

IJES, AWF and TvW carried out the constructions of plasmids and strains and performed silencing assays, western blots and RT-PCR. KV and FF carried out ChIP analyses. BLP performed nuclear localization experiments. TLL performed gene expression profiling and data analysis. IJES, AWF and FvL conceived of the study. SMG and FCPH analyzed the localization and expression data and contributed to the final manuscript. IJES, SMG and FvL wrote the paper. All authors read and approved the final manuscript.

## Supplementary Material

Additional file 1 **Chromatin immunoprecipitation of Sir2 and Sir3 normalized to an actively transcribed gene**. Chromatin immunoprecipitation (ChIP) using specific antibodies against Sir2 and Sir3 [24] was followed by quantitative PCR to determine binding to telomeric *URA3 *and *ACT1 *upon targeting of Dot1 or Dot1^G401R ^(NKI5128). Average ChIP signals were normalized to input levels and Sir protein binding at *URA3 *relative to Sir protein binding at the actively transcribed *ACT1 *locus was plotted (*n *= 2, +/- SEM). Click here for file

Additional file 2 **Characterization of the role of Gcn5 in derepression by Dot1**. **(A) ***URA3 *silencing was determined in strains with a different genomic context at telomere VIIL. The indicated numbers refer to the distance (kb) from the *URA3 *promoter. Strains from top to bottom are NKI1084, NKI1087, NKI5376 and NKI1088. Gene cassettes inserted on the centromeric side of *URA3 *could positively and negatively affect *URA3 *silencing, supporting our observations that silencing is not only determined by linear spreading from the telomeric repeats but can also be influenced by distal sequences. This may help to explain the observed differences in silencing between native telomeres [82]. **(B) **Gcn5 or a catalytic inactive mutant (Gcn5^F221A^) [94] were expressed together with LexA, LexA-Dot1 or LexA-Dot1^G401R ^in a strain lacking endogenous Gcn5 (NKI2214). Under these conditions, expression of Gcn5^F221A ^(and to a lesser extent the empty vector) resulted in slow growth and reduced silencing (for example, see LexA alone). The extremely small colonies on 5FOA plates precluded a reliable analysis of the silencing phenotype. Despite the poor growth conditions of the Gcn5^F221A ^strain, the LexA-Dot1^G401R ^protein consistently allowed colony growth on 5-fluoroorotic acid media, whereas no colonies were observed in the much better-growing *GCN5 *strain. This result indicates that catalytic activity of Gcn5 may be required for derepressor activity of LexA-Dot1 and LexA-Dot1^G401R^. **(C) **Rsc4 is acetylated by Gcn5 and mediates some of the functions of Gcn5 [59]. A barrier assay with LexA-Dot1 and LexA-Dot1^G401R ^revealed that the Dot1 derepressor activity was independent of Rsc4 acetylation at K25. **(D) **Barrier assays in strains lacking the indicated gene. These strains were obtained by crossing the strain containing the tagged telomere with strains of the yeast knockout collection. None of the genes analyzed affected Dot1 derepressor activity. The *gcn5Δ *strain was used as a control. **(E) **Barrier assay in strains in which the indicated genes were deleted by homologous recombination. Because *set1Δ *showed a silencing defect, derepression was examined at 37°C. Click here for file

Additional file 3 **Gcn5 is required for relocalization of ARS1413 to the nuclear interior by Gcn4**. Subnuclear positioning of ARS1413 was monitored in a strain harboring Lac operators next to the origin of replication. The locus was visualized by binding of a GFP-LacI fusion protein to the Lac operators (indicated by green boxes). Subnuclear position was scored relative to the nuclear envelope visualized by a GFP-Nup49 fusion in G1 and S phase cells. Localization data are represented in bar graphs as the percentage of spots in one of three concentric zones of equal surface. The dashed line at 33% corresponds to a random distribution. Spots observed in zone 1 represent ARS1413 localized to the nuclear periphery. The number of cells analyzed is indicated by n. *P *values indicate whether the distributions over the three zones in the cell were significantly different from a random distribution (see Figure 4). ARS1413 showed a non-random distribution with bias towards the nuclear periphery. Insertion of binding sites for the transcriptional activator Gcn4 [95], which is known to recruit and require Gcn5 for its function [96,97], changed the localization of ARS1413 to a more random distribution. Deletion of Gcn5 suppressed the change in localization caused by Gcn4 binding. These results suggest that recruitment of Gcn5 can stimulate the localization of a chromatin domain away from the nuclear periphery. This is in line with the observed role of Gcn5 in derepression of a silenced telomere by the N terminus of Dot1 (Figure 2), which is also involved in relocalization of a tagged telomere away from the nuclear periphery (Figure 4). Click here for file

Additional file 4 **Human Dot1 has derepressor activity in yeast**. **(A) **Human DOT1L (1537 residues) consists of a N-terminal methyltransferase domain homologous to the yeast methyltransferase domain (hDot1^1-340^), followed by a lysine-rich region that shows weak homology to the N-terminal domain of yDot1 (hDot1^318-430^), and a large domain of unknown function [52,98]. The catalytically active N-terminal part of human DOT1L (LexA-hDot1^1-340^), a part containing only the lysine-rich region (LexA-hDot1^318-430^) and the combination of both domains (LexA-hDot1^1-430^) were fused to LexA-V5. **(B) **LexA-tagged hDOT1L proteins were expressed in yeast cells. Protein expression and histone methylation was analyzed as described in Figure 2. LexA fusion proteins of hDot1^1-340 ^and hDot1^1-430 ^showed mono- and dimethylation of H3K79 but no detectable trimethylation in yeast. **(C) **Barrier and desilencing assays (NKI5128 and NKI5376) of LexA-tagged hDOT1L proteins. In strains harboring LexA operators within telomeric heterochromatin, the catalytically active hDOT1L protein showed robust derepressor activity. Although the hDOT1L domain with weak homology to the yeast N-terminal domain was required for the full derepressor activity of hDOT1L (compare hDot1^1-430 ^with Dot1^1-340^), the hDot1^318-430 ^domain alone showed no detectable derepressor activity. These results indicated that the lysine-rich region of human DOT1L is not sufficient for, but contributes to, the derepressor activity in yeast of the conserved methyltransferase domain of DOT1L. Because yeast cells lacking endogenous Dot1 but expressing the hDOT1L methyltransferase domain show no detectable trimethylation, these results also show that efficient trimethylation of H3K79 is not required for derepression. This is in line with previous observations, which showed that multiple levels of H3K79 methylation (that is, mono-, di- and trimethylation) can affect Sir3 binding and silencing [21,27]. Click here for file

Additional file 5 **List of yeast strains used in this study**. Click here for file

Additional file 6 **List of plasmids used in this study**. Click here for file

Additional file 7 **Primer list**. Click here for file
