# Supplementary figures and images for: Dot1 binding induces chromatin rearrangements by histone methylation-dependent and -independent mechanisms
Source: Epigenetics Chromatin. 2011 Feb 3;4:2. doi: 10.1186/1756-8935-4-2 (PMC3038881; doi:10.1186/1756-8935-4-2)

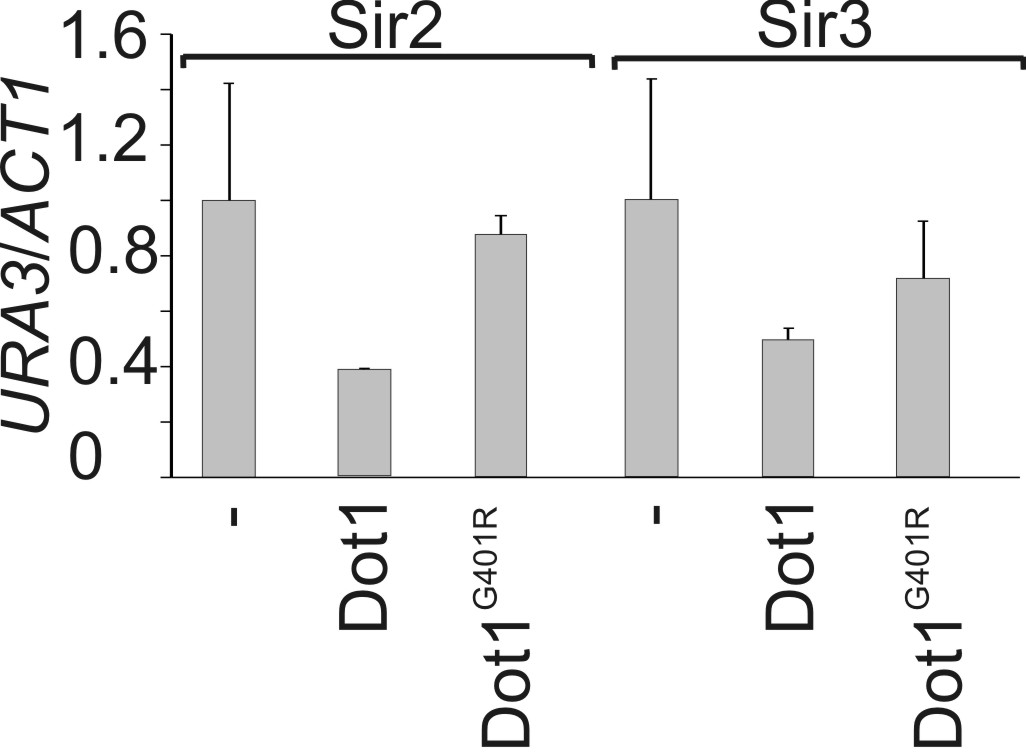

Supplement: Additional file 1 — Chromatin immunoprecipitation of Sir2 and Sir3 normalized to an actively transcribed gene. Chromatin immunoprecipitation (ChIP) using specific antibodies against Sir2 and Sir3 [24] was followed by quantitative PCR to determine binding to telomeric URA3 and ACT1 upon targeting of Dot1 or Dot1G401R (NKI5128). Average ChIP signals were normalized to input levels and Sir protein binding at URA3 relative to Sir protein binding at the actively transcribed ACT1 locus was plotted (n = 2, +/- SEM). [file 1756-8935-4-2-S1.JPEG]

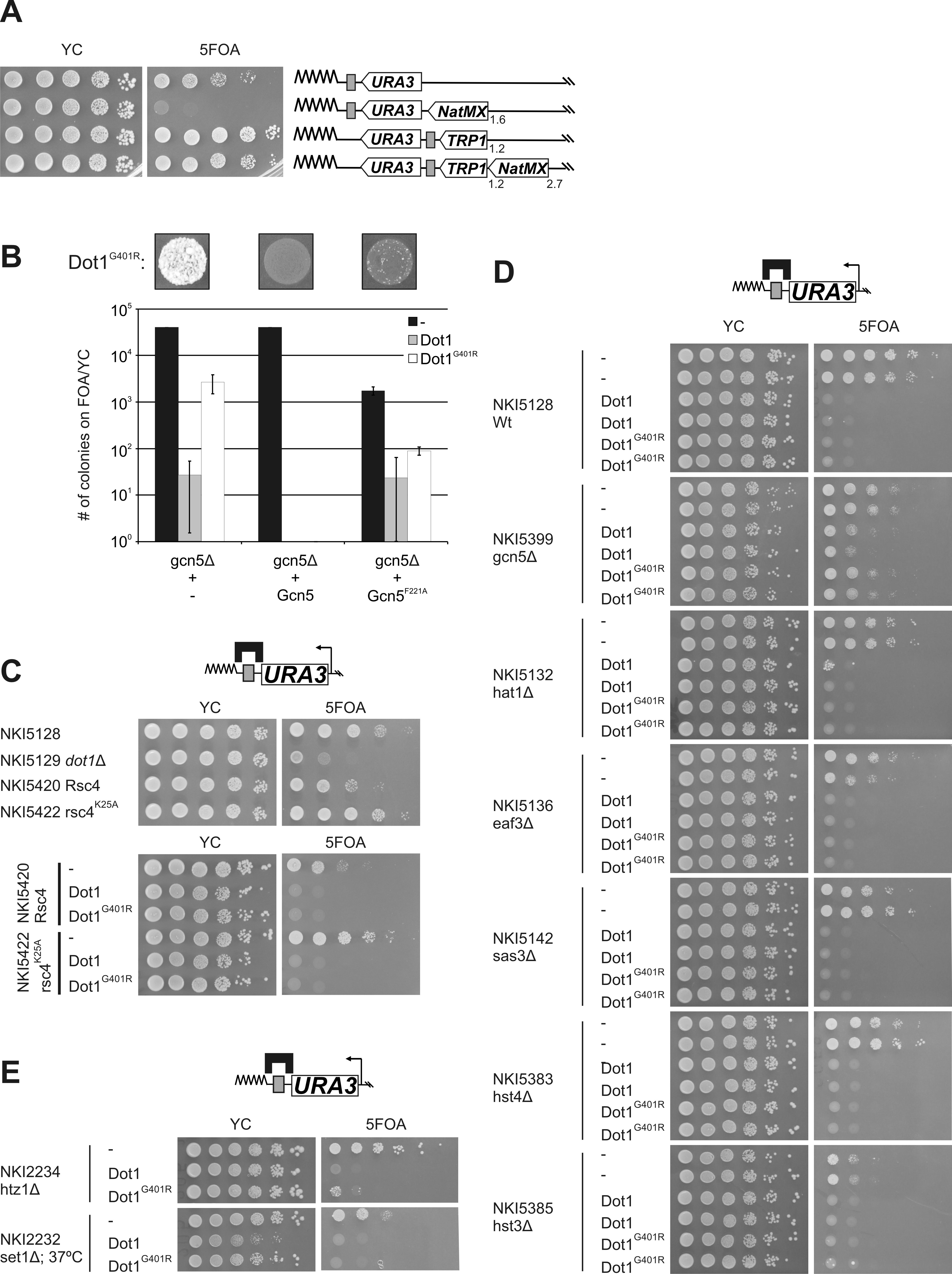

Supplement: Additional file 2 — Characterization of the role of Gcn5 in derepression by Dot1. (A) URA3 silencing was determined in strains with a different genomic context at telomere VIIL. The indicated numbers refer to the distance (kb) from the URA3 promoter. Strains from top to bottom are NKI1084, NKI1087, NKI5376 and NKI1088. Gene cassettes inserted on the centromeric side of URA3 could positively and negatively affect URA3 silencing, supporting our observations that silencing is not only determined by linear spreading from the telomeric repeats but can also be influenced by distal sequences. This may help to explain the observed differences in silencing between native telomeres [82]. (B) Gcn5 or a catalytic inactive mutant (Gcn5F221A) [94] were expressed together with LexA, LexA-Dot1 or LexA-Dot1G401R in a strain lacking endogenous Gcn5 (NKI2214). Under these conditions, expression of Gcn5F221A (and to a lesser extent the empty vector) resulted in slow growth and reduced silencing (for example, see LexA alone). The extremely small colonies on 5FOA plates precluded a reliable analysis of the silencing phenotype. Despite the poor growth conditions of the Gcn5F221A strain, the LexA-Dot1G401R protein consistently allowed colony growth on 5-fluoroorotic acid media, whereas no colonies were observed in the much better-growing GCN5 strain. This result indicates that catalytic activity of Gcn5 may be required for derepressor activity of LexA-Dot1 and LexA-Dot1G401R. (C) Rsc4 is acetylated by Gcn5 and mediates some of the functions of Gcn5 [59]. A barrier assay with LexA-Dot1 and LexA-Dot1G401R revealed that the Dot1 derepressor activity was independent of Rsc4 acetylation at K25. (D) Barrier assays in strains lacking the indicated gene. These strains were obtained by crossing the strain containing the tagged telomere with strains of the yeast knockout collection. None of the genes analyzed affected Dot1 derepressor activity. The gcn5Δ strain was used as a control. (E) Barrier assay in strains in whi [file 1756-8935-4-2-S2.JPEG]

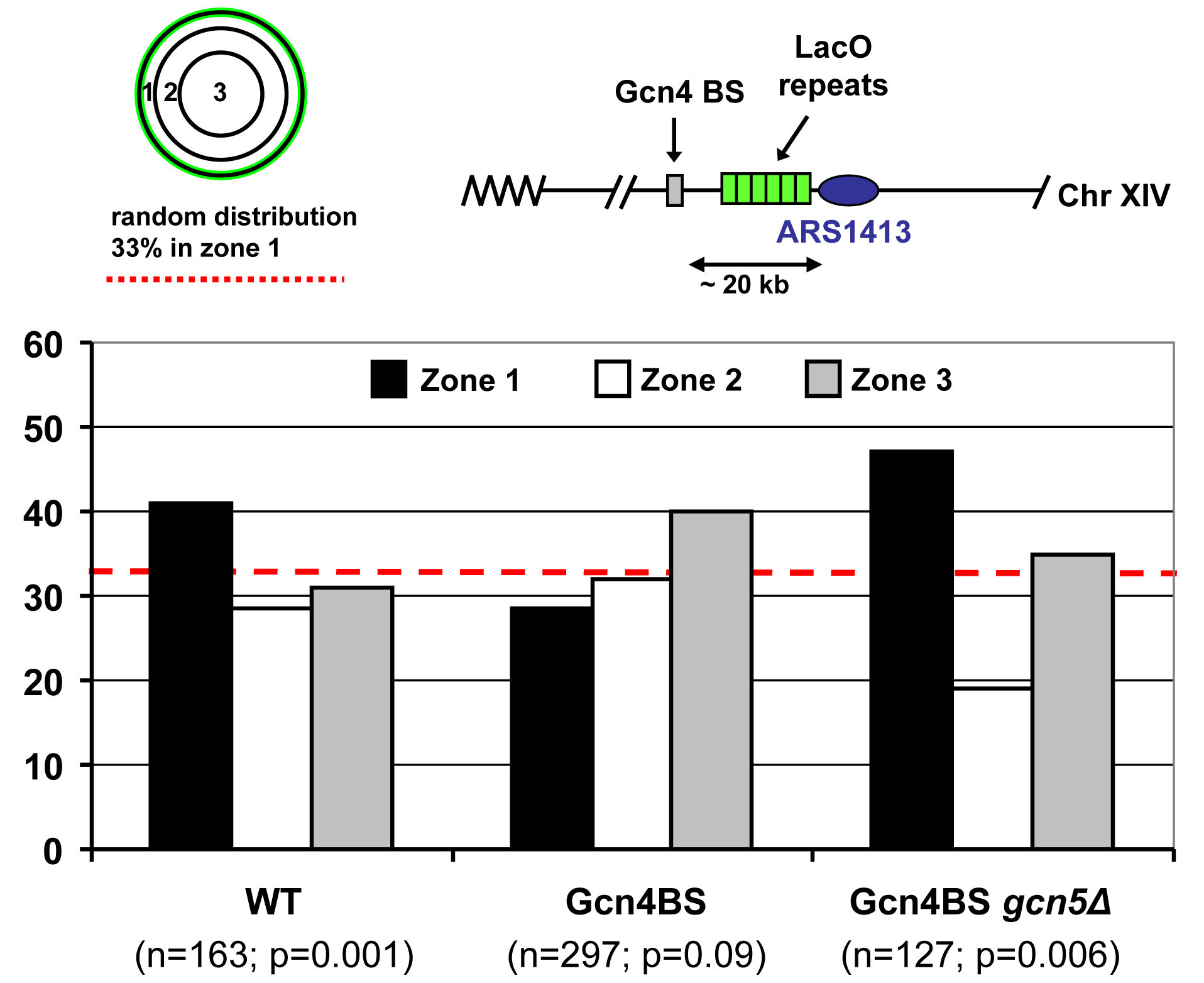

Supplement: Additional file 3 — Gcn5 is required for relocalization of ARS1413 to the nuclear interior by Gcn4. Subnuclear positioning of ARS1413 was monitored in a strain harboring Lac operators next to the origin of replication. The locus was visualized by binding of a GFP-LacI fusion protein to the Lac operators (indicated by green boxes). Subnuclear position was scored relative to the nuclear envelope visualized by a GFP-Nup49 fusion in G1 and S phase cells. Localization data are represented in bar graphs as the percentage of spots in one of three concentric zones of equal surface. The dashed line at 33% corresponds to a random distribution. Spots observed in zone 1 represent ARS1413 localized to the nuclear periphery. The number of cells analyzed is indicated by n. P values indicate whether the distributions over the three zones in the cell were significantly different from a random distribution (see Figure 4). ARS1413 showed a non-random distribution with bias towards the nuclear periphery. Insertion of binding sites for the transcriptional activator Gcn4 [95], which is known to recruit and require Gcn5 for its function [96,97], changed the localization of ARS1413 to a more random distribution. Deletion of Gcn5 suppressed the change in localization caused by Gcn4 binding. These results suggest that recruitment of Gcn5 can stimulate the localization of a chromatin domain away from the nuclear periphery. This is in line with the observed role of Gcn5 in derepression of a silenced telomere by the N terminus of Dot1 (Figure 2), which is also involved in relocalization of a tagged telomere away from the nuclear periphery (Figure 4). [file 1756-8935-4-2-S3.JPEG]

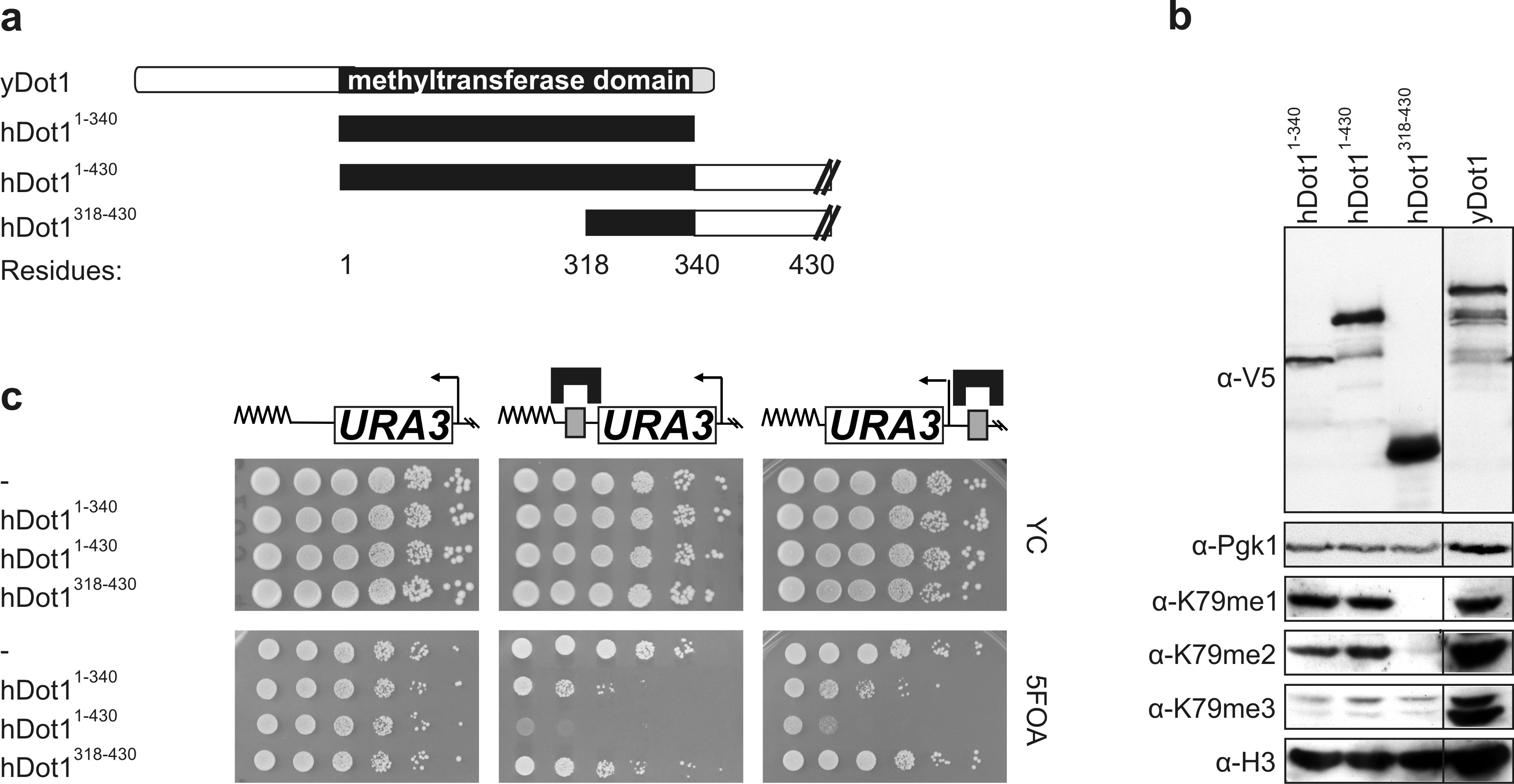

Supplement: Additional file 4 — Human Dot1 has derepressor activity in yeast. (A) Human DOT1L (1537 residues) consists of a N-terminal methyltransferase domain homologous to the yeast methyltransferase domain (hDot11-340), followed by a lysine-rich region that shows weak homology to the N-terminal domain of yDot1 (hDot1318-430), and a large domain of unknown function [52,98]. The catalytically active N-terminal part of human DOT1L (LexA-hDot11-340), a part containing only the lysine-rich region (LexA-hDot1318-430) and the combination of both domains (LexA-hDot11-430) were fused to LexA-V5. (B) LexA-tagged hDOT1L proteins were expressed in yeast cells. Protein expression and histone methylation was analyzed as described in Figure 2. LexA fusion proteins of hDot11-340 and hDot11-430 showed mono- and dimethylation of H3K79 but no detectable trimethylation in yeast. (C) Barrier and desilencing assays (NKI5128 and NKI5376) of LexA-tagged hDOT1L proteins. In strains harboring LexA operators within telomeric heterochromatin, the catalytically active hDOT1L protein showed robust derepressor activity. Although the hDOT1L domain with weak homology to the yeast N-terminal domain was required for the full derepressor activity of hDOT1L (compare hDot11-430 with Dot11-340), the hDot1318-430 domain alone showed no detectable derepressor activity. These results indicated that the lysine-rich region of human DOT1L is not sufficient for, but contributes to, the derepressor activity in yeast of the conserved methyltransferase domain of DOT1L. Because yeast cells lacking endogenous Dot1 but expressing the hDOT1L methyltransferase domain show no detectable trimethylation, these results also show that efficient trimethylation of H3K79 is not required for derepression. This is in line with previous observations, which showed that multiple levels of H3K79 methylation (that is, mono-, di- and trimethylation) can affect Sir3 binding and silencing [21,27]. [file 1756-8935-4-2-S4.JPEG]
