# Supplementary material for: Dot1 binding induces chromatin rearrangements by histone methylation-dependent and -independent mechanisms
Source: Epigenetics Chromatin. 2011 Feb 3;4:2. doi: 10.1186/1756-8935-4-2 (PMC3038881; doi:10.1186/1756-8935-4-2)
Supplement: Additional file 5 — List of yeast strains used in this study. [file 1756-8935-4-2-S5.DOC]

| **Strains** | **Genotype** | **Source** |
| --- | --- | --- |
| NKI1084 | Y7092, URA3-LexO_2_-TEL-VIIL | This study |
| NKI1087 | NKI1084, ADH4terminator::NatMX | This study |
| NKI1088 | Y7092, NatMX-TRP1- LexO_10_-URA3-TEL-VIIL | This study |
| NKI1107 | BY4702, gcn5::kanMX | This study |
| NKI1117 | MATa his3Δ200 leu2Δ0 met15Δ0 trp1Δ63 ura3Δ0 URA3-LexO_3_-TEL-VIR | This study |
| NKI1118 | MATa his3Δ200 leu2Δ0 met15Δ0 trp1Δ63 ura3Δ0 TRP1-LexO_10_-URA3-TEL-VIR | This study |
| NKI2229 | Y7092, LexO_5_-URA3-TEL-XIL | This study |
| NKI2230 | Y7092, LexO_5_-URA3-TEL-XVIL | This study |
| NKI2231 | Y7092, LexO_5_-URA3-TEL-XVR | This study |
| NKI2232 | NKI5128, MATa, set1::KanMX | This study |
| NKI2234 | NKI5128, MATa, htz1::KanMX | This study |
| NKI5070 | Y7092, dot1::NatMX , URA3-TEL-VIIL | This study |
| NKI5072 | Y7092, dot1::NatMX , URA3-lexO_3_-TEL-VIIL | This study |
| NKI5128 | Y7092, MATa URA3-LexO_3_-TEL-VIIL | This study |
| NKI5129 | Y7092, MATa dot1::NatMX, URA3-LexO_3_-TEL-VIIL | This study |
| NKI5132 | NKI5072, MATa, hat1::KanMX | This study |
| NKI5136 | NKI5072, MATa, eaf3::KanMX | This study |
| NKI5142 | NKI5072, MATa, sas3::KanMX | [1] |
| NKI5240 | Y7092, MATa URA3-TEL-VIIL | This study |
| NKI5376 | Y7092, TRP1-LexO_10_-URA3-TEL-VIIL | This study |
| NKI5378 | Y7092, dot1::NatMX, TRP1- LexO_10_-URA3-TEL-VIIL | This study |
| NKI5383 | NKI5072, MATa, hst4::KanMX | [1] |
| NKI5385 | NKI5072, MATa, hst3::KanMX | [1] |
| NKI5399 | NKI5128, gcn5::KanMX | This study |
| NKI5420 | YBC2898, URA3- LexO_3_-TEL-VIIL | This study |
| NKI5422 | YBC2899, URA3- LexO_3_-TEL-VIIL | This study |
| NKI6018 | NKI1088, gcn5::KanMX | This study |
| NKI6020 | NKI1088, dot1::HphMX | This study |
| BY4702 | MATα leu2Δ0 lys2Δ0 met15Δ0 | [2] |
| GA-1459 | MATa ade2-1 can1-100 his3-11,15::GFP-LacI-HIS3 trp1-1 ura3-1 leu2-3,112 nup49::NUP49-GFP-URA3 TEL-VIR::lacO-lexAop-TRP1 | [3] |
| L40 | MATa his3Δ200, trp1-901, leu2-3,112, ade2, LYS2::LYS2- LexO_4_-HIS3 URA3::URA3- LexO_8_-LacZ | [4] |
| BY4727 | MATα his3Δ200 leu2Δ0 lys2Δ0 met15Δ0 trp1Δ63 ura3Δ0 | [2] |
| UCC1369 | MATa ade2Δ::hisG his3Δ200 leu2Δ0 lys2Δ0 met15Δ0 trp1Δ63 ura3Δ0 adh4::URA3-TEL-VIIL ADE2-TEL-VR hhf2-hht2Δ::MET15 hhf1-hht1Δ::LEU2 pMP9 (H3) | [5] |
| NKI1043 | MATa his3Δ200 leu2Δ0 lys2Δ0 met15Δ0 trp1Δ63 ura3Δ0 hhf2-hht2Δ::MET15 hhf1-hht1Δ::LEU2 pMP9 (H3) | This study |
| NKI6041 | MATa his3Δ200 leu2Δ0 lys2Δ0 met15Δ0 trp1Δ63 ura3Δ0 hhf2-hht2Δ::MET15 hhf1-hht1Δ::leu2::HIS3 pMP9 (H3, LYS+)) | This study |
| NKI6042 | NKI6041, URA3- LexO_3_-TEL-VIIL | This study |
| NKI6043 | NKI6041, URA3-TEL-VIIL | This study |
| NKI6045 | NKI6042, pMP3 (H3, TRP+) | This study |
| NKI6047 | NKI6042, pFvl88 (H3K79R, TRP+) | This study |
| NKI6049 | NKI6043, pMP3 (H3, TRP+) | This study |
| NKI6051 | NKI6043, pFvl88 (H3K79R, TRP+) | This study |
| YBC2898 | MATα lys2Δ0 met15Δ0 his3Δ1 leu2Δ0 ura3Δ0 RSC4 | [6] |
| YBC2899 | MATα lys2Δ0 met15Δ0 his3Δ1 leu2Δ0 ura3Δ0 RSC4^K25A^ | [6] |
| YXB85-n | W303 E-HMLalpha-I::E-HMLalpha-I(inverted)-URA3 | [7] |
| YQY10 | W303 E-HMLalpha-I::E-HMLalpha-I(inverted)- LexO_2_-URA3 | [7] |
| YQY09 | W303 E-HMLalpha-I::E-HMLalpha-I(inverted)-URA3- LexO_2_ | [7] |
| Y7092 | MATα can1Δ::STE2pr-Sp_his5 lyp1Δ his3Δ1 leu2Δ0 ura3Δ0 met15Δ0 | [8] |
| BY4733 | MATa his3Δ200 leu2Δ0 met15Δ0 trp1Δ63 ura3Δ0 | [2] |
| NKI3006 | MATα his3Δ1 leu2Δ0 lys2Δ0 ura3Δ0 dot1::URA3 | [9] |

1. Verzijlbergen KF, Faber AW, Stulemeijer IJE, van Leeuwen F: **Multiple histone modifications in euchromatin promote heterochromatin formation by redundant mechanisms in *Saccharomyces cerevisiae*.** *BMC Molecular Biology* 2009, **10**.

2. Brachmann CB, Davies A, Cost GJ, Caputo E, Li J, Hieter P, Boeke JD: **Designer deletion strains derived from *Saccharomyces cerevisiae* S288C: a useful set of strains and plasmids for PCR-mediated gene disruption and other applications.** *Yeast* 1998, **14:**115-132.

3. Taddei A, Van Houwe G, Hediger F, Kalck V, Cubizolles F, Schober H, Gasser S: **Nuclear pore association confers optimal expression levels for an inducible yeast gene.** *Nature* 2006, **441:**774-778.

4. Vojtek A, Hollenberg S, Cooper J: **Mammalian Ras interacts directly with the serine/threonine kinase raf.** *Cell* 1993, **74:**205-214.

5. van Leeuwen F, Gafken PR, Gottschling DE: **Dot1p modulates silencing in yeast by methylation of the nucleosome core.** *Cell* 2002, **109:**745-756.

6. VanDemark A, Kasten M, Ferris E, Heroux A, Hill C, Cairns B: **Autoregulation of the Rsc4 tandem bromodomain by Gcn5 acetylation.** *Mol Cell* 2007, **27:**817-828.

7. Chiu YH, Yu Q, Sandmeier JJ, Bi X: **A targeted histone acetyltransferase can create a sizable region of hyperacetylated chromatin and counteract the propagation of transcriptionally silent chromatin.** *Genetics* 2003, **165:**115-125.

8. Tong AH, Boone C: **Synthetic genetic array analysis in *Saccharomyces cerevisiae*.** *Methods Mol Biol* 2006, **313:**171-192.

9. Frederiks F, Tzouros M, Oudgenoeg G, van Welsem T, Fornerod M, Krijgsveld J, van Leeuwen F: **Nonprocessive methylation by Dot1 leads to functional redundancy of histone H3K79 methylation states.** *Nat Struct Mol Biol* 2008, **15:**550-557.
