# Supplementary material for: Dot1 binding induces chromatin rearrangements by histone methylation-dependent and -independent mechanisms
Source: Epigenetics Chromatin. 2011 Feb 3;4:2. doi: 10.1186/1756-8935-4-2 (PMC3038881; doi:10.1186/1756-8935-4-2)
Supplement: Additional file 6 — List of plasmids used in this study. [file 1756-8935-4-2-S6.DOC]

| **Plasmids** | **Comments** | **Source** |
| --- | --- | --- |
| pRS425 | 2μ-LEU2 | [1] |
| pBTM116 | 2μ-TRP1-PADH1-LexA | [2] |
| pLexA-Lamin | 2μ-TRP1-PADH1-LexA-Lamin | [2] |
| pLexA-MCycE | 2μ-TRP1-PADH1-LexA-CyclinE-mut | [3] |
| pFvL23 | 2μ-TRP1-PADH1-LexA-Dot1 | This study |
| pL3 (pLexA-Adr1^415-467^) | 2μ-LEU2-PADH1-LexA-ADR1-AD (415-467) | [4] |
| pRQ12 (pLexA-Gcn5) | 2μ-LEU2-PADH1-LexA-GCN5 | [4] |
| pFvL232 | 2μ-LEU2-PADH1-LexA-V5 | This study |
| pFvL909 | 2μ-LEU2-PADH1-LexA-V5-NLS | This study |
| pFvL230 | 2μ-LEU2-PADH1-LexA-V5-Dot1 | This study |
| pFvL908 | 2μ-LEU2-PADH1-LexA-V5-Dot1^G401R^ | This study |
| pFvL905 | 2μ-LEU2-PADH1-LexA-V5-Dot1^1-237^ | This study |
| pFvL913 | 2μ-LEU2-PADH1-LexA-V5-Dot1^1-172^ | This study |
| pFvL901 | 2μ-LEU2-PADH1-LexA-V5-NLS-Dot1^172-582^ | This study |
| pIS001 | 2μ-LEU2-PADH1-LexA-V5-Dot1-NLS-Dot1^172-582 G401R^ | This study |
| pFvL921 | 2μ-LEU2-PADH1-LexA-V5-RPD3 | This study |
| pFvL250 | 2μ-LEU2-PADH1-LexA-V5-Ecm5-HA-TAP | This study |
| pFvL925 | 2μ-LEU2-PADH1-LexA-V5-hDot1^1-430^ | This study |
| pFvL927 | 2μ-LEU2-PADH1-LexA-V5-hDot1^318-430^ | This study |
| pRS315 | CEN-LEU2-PADH1 | [5] |
| pFvL914 | CEN-LEU2-PADH1-LexA-V5-Dot1 | This study |
| pFvL916 | CEN-LEU2-PADH1-LexA-V5-Dot1^G401R^ | This study |
| pMP3 | CEN-TRP1-HHF2-HHT2 | [6] |
| pFvL87 | CEN-TRP1-HHF2-HHT2-K79A | [6] |
| pFvL88 | CEN-TRP1-HHF2-HHT2-K79R | This study |
| pRS414 Gcn5 | CEN-TRP1-GCN5 | [7] |
| pRS414 Gcn5 F221A | CEN-TRP1-GCN5^F221A^ | [7] |
| pRS305 | CEN-LEU2 | [5] |
| pYMR176 | 2μ-URA3-pGAL-MORF-HIS-HA-TAP | Open Biosystems |
| pFvl99 | 2μ-NatMX | This study |
| pFvl100 | 2μ-HphMX | This study |
| pRS400 | 2μ-KanMX | [5] |
| pAG25 | 2μ-pTEF-NatMX-tTEF | [8] |
| pAG32 | 2μ-pTEF-HphMX-tTEF | [8] |
| pVIIL-URA3-LexAS3-TEL | URA3-LexO_3_-TEL-VIIL | [9] |
| pVIIL-URA3-LexAS2-TEL | URA3-LexO_2_-TEL-VIIL | [9] |
| pADH4UCA-IV | URA3-TEL-VIIL | [10] |
| pT7 | LexO_10_-URA3-TEL-VIIL | [4] |

1. Christianson T, Sikorski R, Dante M, Shero J, Hieter P: **Multifunctional yeast high-copy-number shuttle vectors.** *Gene* 1992, **110:**119-122.

2. Bartel P, Chien CT, Sternglanz R, Fields S: **Elimination of false positives that arise in using the two-hybrid system.** *Biotechniques* 1993, **14:**920-924.

3. Singer J, Gurian-West M, Clurman B, Roberts J: **Cullin-3 targets cyclin E for ubiquitination and controls S phase in mammalian cells.** *Genes Dev* 1999, **13:**2375-2387.

4. Bi X, Yu Q, Sandmeier JJ, Zou Y: **Formation of boundaries of transcriptionally silent chromatin by nucleosome-excluding structures.** *Mol Cell Biol* 2004, **24:**2118-2131.

5. Sikorski RS, Hieter P: **A system of shuttle vectors and yeast host strains designed for efficient manipulation of DNA in *Saccharomyces cerevisiae*.** *Genetics* 1989, **122:**19-27.

6. van Leeuwen F, Gafken PR, Gottschling DE: **Dot1p modulates silencing in yeast by methylation of the nucleosome core.** *Cell* 2002, **109:**745-756.

7. van Oevelen CJ, van Teeffelen HA, van Werven FJ, Timmers HT: **Snf1p-dependent Spt-Ada-Gcn5-acetyltransferase (SAGA) recruitment and chromatin remodeling activities on the HXT2 and HXT4 promoters.** *J Biol Chem* 2006, **281:**4523-4531.

8. Goldstein AL, McCusker JH: **Three new dominant drug resistance cassettes for gene disruption in *Saccharomyces cerevisiae*.** *Yeast* 1999, **15:**1541-1553.

9. Lustig AJ, Liu C, Zhang C, Hanish JP: **Tethered Sir3p nucleates silencing at telomeres and internal loci in *Saccharomyces cerevisiae*.** *Mol Cell Biol* 1996, **16:**2483-2495.

10. Gottschling DE, Aparicio OM, Billington BL, Zakian VA: **Position effect at *S. cerevisiae* telomeres: reversible repression of Pol II transcription.** *Cell* 1990, **63:**751-762.
