# Supplementary material for: Dot1 binding induces chromatin rearrangements by histone methylation-dependent and -independent mechanisms
Source: Epigenetics Chromatin. 2011 Feb 3;4:2. doi: 10.1186/1756-8935-4-2 (PMC3038881; doi:10.1186/1756-8935-4-2)
Supplement: Additional file 7 — Primer list. [file 1756-8935-4-2-S7.DOC]

| **Oligonucleotides** | **Sequence** | **Used for:** |
| --- | --- | --- |
| URA3orf_Qfor | GGGCAGACATTACGAATGCA | qPCR *URA3* |
| URA3orf_Qrev | CCTGCTTCAAACCGCTAACAA | qPCR *URA3* |
| HMLup_Qfor | TTGGCTCCGGTGTAAAACAAA | qPCR *HMLα* |
| QHMLup_Qrev | CCGCGTGCCATTCTTCAG | qPCR *HMLα* |
| ACT1_Qfor | CTCTTTTTATCTTCCTTTTTTTCCTCTCT | qPCR *ACT1* |
| ACT1_Qrev | CGTGAAAAATCTAAAAGCTGATGTAGTAG | qPCR *ACT1* |
| midURA3+ | CATTACGAATGCACACGGTG | RT-PCR *URA3* |
| URA3mid2 | TCCACCCATGTCTCTTTGAG | RT-PCR *URA3* |
| Sir3-1 | GACGAACAGTCAAGCTTGGAC | RT-PCR *SIR3* |
| Sir3-2 | TGCATCCATTCCTGCCAGTTC | RT-PCR *SIR3* |
| URA3_TELVIRtr_F1 | TCCTCGGACAGTTCAACTCATCCATGATTGTTCTAGAGGTGACCAAGCTTTTCAATTCAATTCATC | integration |
| URA3_TELVIRtr_F2 | TCCTCGGACAGTTCAACTCATCCATGATTGTTCTAGAGGTGTCTTTACCGTCTTTGTATGC | integration |
| URA3_TELVIRtr_R1 | AGGGTTTTCCCAGTCACGACGTTG | integration |
| TELVIR-3907_F | AAGAAGAACGCTAATACTCGT | integration |
| TELVIR-3463_R | CTCTAGAACAATCATGGATGAG | integration |
| LUJ1 | GGTAGAATTTGACACAAATGAAGACGAACGATGGCTTTTCAATTCATC | integration |
| LUJ2 | GATGAATTGAAAAGCCATCGTTCGTCTTCATTTGTGTCAAATTCTACC | integration |
| BDUL-11L-R1 | TTATTGTTGATAGAACACTAACCCTTCAGCTTTATTTCTGGTTACCTCACATCTACCTCTACTCTG | integration |
| DLU-11L-DF2 | CTGAAAATATCAAAATTTCTGGGTTGCGATAGTTTTTGTGTAACCAAGAAAAAGGGGAACTATTACG | integration |
| BDUL-15R/16L-R1 | AAATATTCCATTCTTCAACAATAATACATAAACATATTGACTTCTCACATCTACCTCTACTCTG | integration |
| DLU-15R/16L-DF2 | ATTAAGGAACTTTTACGTTAATGACGTCATGGTAGTGCTCGTACAAGAAAAAGGGGAACTATTACG | integration |
| ADH4tINT1KO | CGAACGAACTCATAAACGTCAATTATGCGTGTGCCTTATTGATTGTACTGAGAGTGCACC | integration |
| ADH4tINT2KO | GATTGGTAAATAAATAAGTGCATTATACTGTACGCACAACCTGTGCGGTATTTCACACCG | integration |
| LexADot1V5P2 | CGTAGAATCGAGACCGAGGAGAGGGTTAGGGATAGGCTTACCCAGCCAGTCGCCGTTGCGA | plasmid |
| LexAV5P3 | GGTAAGCCTATCCCTAACCCTCTCCTCGGTCTCGATTCTACGGAATTCCCGGGGATCCGTCGA | plasmid |
| LexADot1V5P3 | GGTAAGCCTATCCCTAACCCTCTCCTCGGTCTCGATTCTACGGAATTCGGCGGTCAAGAAAG | plasmid |
